# Correction: Sokołowski et al. Immune Checkpoint Inhibitors (ICI) in Urological Cancers: A New Modern Era, but Not Generally Applied. *Int. J. Mol. Sci.* 2025, *26*, 7194

**DOI:** 10.3390/ijms26188993

**Published:** 2025-09-16

**Authors:** Marcin Sokołowski, Anna Sokołowska, Magdalena Chrząszcz, Aleksandra Butrym

**Affiliations:** 1 Dr Alfred Sokołowski Specialist Hospital in Walbrzych, 58-309 Walbrzych, Poland; annabiernacka7@gmail.com (A.S.); lek.mmch@gmail.com (M.C.); aleksandra.butrym@gmail.com (A.B.); 2 Lower Silesian Oncology, Pulmonology and Hematology Center, pl. Hirszfelda 12, 53-413 Wroclaw, Poland; 3 Department of Cancer Prevention and Therapy, Wroclaw Medical University, ul Borowska 2-13, 50-556 Wroclaw, Poland

In the original publication [1], there was a mistake in the Funding section of this review. The authors would like to change the Funding information from “This research was funded by supported by a grant of the Medical Research Agency KPOD.07.07-IW.07-0321/24.” to “This research received no external funding.” The authors state that the scientific conclusions are unaffected. This correction was approved by the Academic Editor. The Funding section should be corrected as follows.
